# Expression of DNA repair and cell cycle control genes in HPV infection

**DOI:** 10.18699/vjgb-25-46

**Published:** 2025-06

**Authors:** E.V. Mashkina, V.V. Volchik, E.S. Muzlaeva, E.G. Derevyanchuk

**Affiliations:** Southern Federal University, Rostov-on-Don, Russia; Southern Federal University, Rostov-on-Don, Russia; Southern Federal University, Rostov-on-Don, Russia; Southern Federal University, Rostov-on-Don, Russia

**Keywords:** human papillomavirus, cell cycle control, gene transcription, coexpression genes, вирус папилломы человека, контроль клеточного цикла, транскрипция генов, коэкспрессия генов

## Abstract

One of the main etiological factors in the development of cervical cancer is infection with human papillomavirus (HPV). At the same time, the risk of developing a malignant process increases with an increase in viral load. The aim of this study was to investigate the transcription level of DNA repair and cell cycle control genes in the cervical epithelial cells of women with a clinically significant HPV viral load. The material for the study was DNA and RNA samples isolated from cervical epithelial cells in women. A total of 107 samples were analyzed. 55 women were HPV-positive (with a clinically significant viral load – more than 103 HPV genomes per 100 thousand human cells); the control group consisted of 52 HPV-negative women. All women were over 30 years old. The transcription level of the APEX1, ERCC2, CHEK2, TP53, TP73, CDKN2A, SIRT1 genes was determined using RT-PCR. It was shown that the detection frequency of the APEX1 and ERCC2 gene transcripts was increased in the group of women with a clinically significant viral load. The transcription level of all the studied genes did not differ between the control group and the group with clinically significant HPV concentrations. However, the transcription level of the TP53 and TP73 genes decreased with increasing viral load. In the control, a correlation between the transcription levels of genes involved in the functioning of the p53 protein was revealed. An increase in viral load during HPV infection is associated with a change in the coexpression of DNA repair and cell cycle control genes.

## Abstract (ru)

Одним из основных этиологических факторов развития рака шейки матки является инфицирование вирусом папилломы человека (ВПЧ). При этом риск развития злокачественного процесса увеличивается с ростом вирусной нагрузки. Целью данного исследования было изучение уровня транскрипции генов репарации ДНК и контроля клеточного цикла в эпителиальных клетках цервикального канала женщин с клинически значимой вирусной нагрузкой при ВПЧ-инфекции. Материалом для исследования послужили образцы ДНК и РНК, выделенные из эпителиальных клеток. Всего было проанализировано 107 образцов: 55 женщин были ВПЧ-положительными (с клинически значимой вирусной нагрузкой – более 103 геномов ВПЧ на 100 тыс. клеток человека), контрольную группу составили 52 ВПЧ-отрицательные женщины. Все женщины были старше 30 лет. Уровень транскрипции генов APEX1, ERCC2, CHEK2, TP53, TP73, CDKN2A, SIRT1 определяли с помощью ОТ-ПЦР. Показано, что частота обнаружения транскриптов генов APEX1 и ERCC2 была повышена в группе женщин с клинически значимой вирусной нагрузкой. Уровень транскрипции всех исследованных генов не различался между контрольной группой и группой ВПЧ-инфицированных женщин. Однако установлено, что уровень транскрипции генов TP53 и TP73 снижается с ростом вирусной нагрузки. В контроле выявлена корреляция между уровнями транскрипции генов, участвующих в обеспечении функционирования белка p53. Увеличение вирусной нагрузки при инфицировании ВПЧ связано с изменением коэкспрессии генов репарации ДНК и контроля клеточного цикла.

## Introduction

Human papillomavirus (HPV) is a major risk factor for invasive cervical cancer (Bava et al., 2016). Most cases of HPV infections (70–80 %) are transient and asymptomatic, in which the virus disappears from the body or becomes undetectable within two years of infection (Kim et al., 2012). However, in some cases, infected women develop low- and high-grade squamous intraepithelial lesions or cervical cancer (Chansaenroj et al., 2013). The International Agency for Research on Cancer has documented that a high viral load in HPV infection is a risk factor for cervical cancer (Ylitalo et al., 2000; van der Weele et al., 2016).

The life cycle of human papillomavirus is determined by its influence on intracellular processes, primarily cell cycle control, the activity of factors of the DNA repair system, and factors of the immune system. Normally, differentiation of urogenital tract epithelial cells is accompanied by their exit from the cell cycle. In infected cells, viral DNA is amplified to thousands of copies per cell, which is accompanied by the preservation of the ability of epithelial cells to divide (Longworth, Laimins, 2004; Münger et al., 2004).

Infection of epithelial cells and amplification of viral DNA can lead to damage to the human genome. The response to DNA damage plays a crucial role in maintaining genome stability by coordinating the course of the cell cycle with DNA repair. The main stages of excision repair include recognition of DNA damage, unwinding of the DNA region with a damaged site (ERCC2 is one of the participants), cutting of the DNA chain and cutting out the damaged DNA region (for example, with the participation of APX1), ligation of a new chain. The implementation of repair processes requires stopping the cell cycle, which is carried out due to the functioning of proteins such as CHEK2, CDKN2A, p53, p73, SIRT1.

Aberrant expression of key factors changes the capacity of the repair system, affecting genome stability and integrity, which increases the likelihood of altered/damaged cells preservation and the development of a malignant process (Kushwah et al., 2023). Research data show that the expression level of the DNA repair and cell cycle control systems genes changes in cervical cancer, including that of APEX1 (Li et al., 2021; Zhang et al., 2023), ERCC2 (Ye et al., 2012; Bajpai et al., 2013), TP53 (Ngan et al., 2001; Zhou et al., 2015), TP73 (Liu et al., 2004; Choi et al., 2007), CDKN2A (Hafkamp et al., 2009). However, there are almost no data on the effect of clinically significant viral load in HPV infection on transcription of the repair system genes before the development of severe forms of epithelial cell dysplasia.

The aim of this study was to investigate the expression level of the DNA repair and cell cycle control systems genes at a clinically significant concentration of human papillomavirus in the epithelial cells of the urogenital tract of women.

## Materials and methods

The material for the study was DNA and RNA samples isolated from epithelial cells of the urogenital tract of women. Among them, there were 55 women infected with HPV (with a clinically significant viral load – more than 3 lg (lg – common logarithm, 3 lg – 1000 HPV genomes per 100 thousand cells)) and 52 HPV-negative women (control group). The average viral load in infected women was 5.03 ± 0.13 lg (minimum – 3.4 lg, maximum – 8.1 lg). Women with a viral load less than 3 lg were excluded from the study.

Among 55 women infected with HPV, 5.4 % had normal cytological characteristics of the epithelium (NILM); 36.4 % had atypical squamous cells of undetermined significance (ASCUS); low-grade dysplasia (LSIL) was detected in 58.2 % of women.

All women included in the study were over 30 years old. The average age of women in the control group was 37 ± 0.79 years, in the group of women with clinically significant HPV concentrations – 38 ± 1.25 years. All collected samples of epithelial cells scrapings from the urogenital tract of the women were provided by the diagnostic laboratory “Nauka” (Rostov-on- Don, Russia).

The ethnic component was also considered – only women of the Caucasian race were included in the study. In this case, Russians made up 86 %, Armenians, 9 %, other nationalities of the Caucasian race, 5 %.

Informed written consent was obtained from all women. The research received approval from the Bioethics Committee of the Academy of Biology and Biotechnology of the Southern Federal University on March 29, 2016 (Protocol No. 2). All experimental procedures adhere to the standards and ethical guidelines of the World Medical Association (Helsinki Declaration).

Samples of cervical epithelial cells scrapings were used. Total DNA was extracted using the DNA-sorb-AM reagent kit according to protocol (NextBio, Russia). DNA for highrisk HPV types (16, 18, 31, 33, 35, 39, 45, 51, 52, 56, 58, and 59) was quantified using the protocol AmpliSens-HPV HCR screen-titre-FRT (Interlabservice, Russia) (AmpliSens… Manual, 2018). An assessment of viral load was made based on clinical reports published in the manufacturer’s kit manual. Specifically, a viral load less than 3 lg per 105 human cells was taken to signify “low clinical significance”, while a load greater than 3 lg per 105 human cells was equated to a clinically significant probability of dysplasia.

Total RNA isolation was performed using the GeneJET RNA Purification Kit (Thermo Scientific) according to the manufacturer’s instructions. The reverse transcription reaction was performed at 45 °C for 50 minutes followed by MMLV-RT inactivation at 92 °C for 5 minutes. The transcription level of the GAPDH, APEX1, ERCC2, CHEK2, TP53, TP73, CDKN2A, SIRT1 genes in epithelial cells of the cervical canal of the women was determined by Real-time PCR using fluorescent gene-specific probes or SYBR Green. The sequence of primers and probes is presented in Table 1. The amplification reaction was performed in two replicates for each sample according to the following program: 94 °C – 10 minutes, 60 °C – 50 seconds, 94 °C – 15 seconds. The last two steps were repeated 40 times.

**Table 1.**
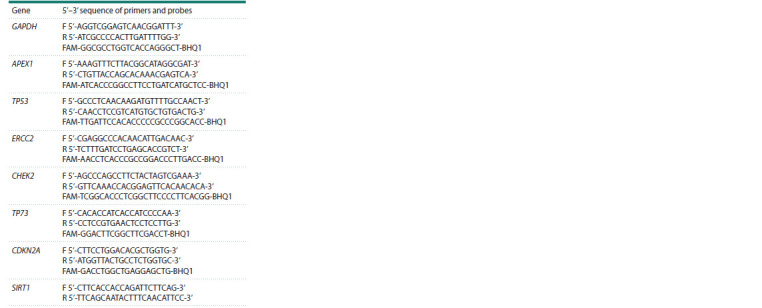
Sequence of primers and probes used to determine the level of gene transcription

Statistical analysis. The target mRNA level was normalized to the level of mRNA GAPDH. The level of transcription was determined using the ΔCt approach, where ΔCt is the difference in the target and the housekeeping gene threshold cycles. Statistical analysis of data of gene expression was performed by the 2–ΔΔCt method by Livаk and Schmittgen (2001). It shows the multiplicity of changes in gene expression level in the samples compared. The average ΔCt values for two groups were compared using Student’s t-test. Pearson’s Rank Correlation Coefficient was used to assess whether there were statistical correlations between the expression level and the selected independent variables (the viral load and women’s age). GraphPad InStat software (version 3.05) was used for all statistical analyses.

## Results

Among 55 women infected with high-risk HPV types, type 16 was detected in 32.7 % of cases. HPV type 18 was the second most frequently detected type (9.1 %). Women infected with either type 31 or type 33 accounted for 7.3 % each. HPV type 51 was detected in three women (5.4 %). In addition, isolated cases of monoinfection with HPV types 45, 52, 58 or 59 were also detected. However, 29.1 % of women were co-infected with two HPV types. No relationship was found between the type of human papillomavirus and the viral load, as well as the transcription level of the studied protein-coding genes.

As a result of the study, it was found that TP53 and SIRT1 transcripts are detected in 100 % of the samples; CHEK2 transcripts are detected in 96 % of the samples. Most samples were characterized by transcription of TP73 (69 and 73 % in the control and HPV infection groups) and CDKN2A (58 and 62 % in the control and HPV infection groups). It was found that in a clinically significant viral load, the part of epithelial cell samples with transcribed APEX1 and ERCC2 genes was 78 and 40 %, respectively, which is 20 % higher than in the control group ( p < 0.05). It should be noted that for the ERCC2 gene, the transcript detection frequency increased with increasing viral load. So, at an HPV concentration of 3–5 lg, the proportion of samples with ERCC2 transcripts was 34.4 %, and at a viral load above 5 lg, it was 47.8 %. This may reflect an increase in the intensity of human DNA damage when the HPV concentration in epithelial cells increases. The highest level of transcription was typical of the CHEK2 and SIRT1 genes.

**Fig. 1.**
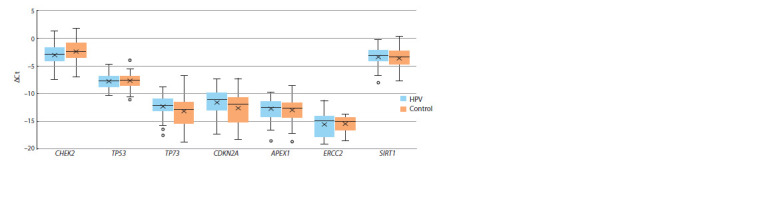
The level of the genes’ transcription in the epithelial cells of the urogenital tract of women relative to the expression of GAPDH in the control and at clinically significant HPV concentrations.

The transcription level of all the genes studied in the control and at clinically significant HPV concentrations did not differ ( p > 0.05) (Fig. 1).

**Fig. 2.**
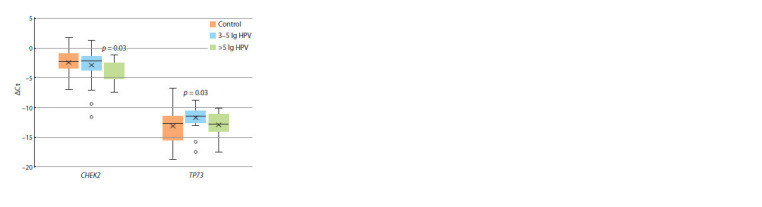
The level of CHEK2 and TP73 transcription in the epithelial cells of the urogenital tract of women relative to the expression of GAPDH depending on the viral load in HPV infection (comparison with the corresponding control).

However, the mRNA level of two genes depended on HPV concentrations in the cells of infected women. Thus, the transcript level of the СНЕK2 gene in the cells of women with a viral load of 3–5 lg did not differ from the control. At the same time, at HPV concentrations of more than 5 lg, the relative level of CHEK2 transcripts was 3 times lower compared to the control (2–ΔΔCt = 0.34, p = 0.03). Another dependence was revealed for the TP73 gene: with a viral load of 3–5 lg HPV genomes, the level of TP73 transcripts was 2.7 times higher than in the control ( p = 0.03); with a viral load of more than 5 lg, the level of TP73 gene transcripts did not differ from the control values (Fig. 2).

A negative correlation was found between the mRNA level of TP73 or TP53 and the viral load in HPV infection (Table 2).

**Table 2.**
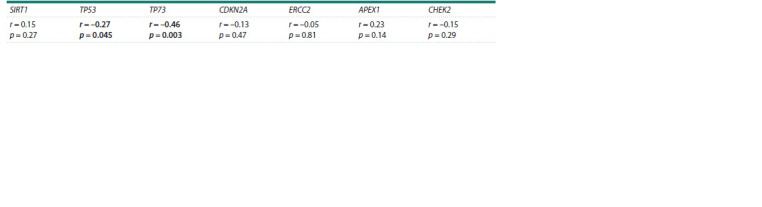
Correlation analysis of the genes’ transcription level with viral load in HPV infection

In the control, the transcription level of CHEK2 correlates with the transcription levels of SIRT1, TP53 and TP73 (Table 3). The level of SIRT1 transcripts correlates with the mRNA levels of the TP53, TP73 and APEX1 genes. TP53 transcription activity is also associated with CDKN2A transcription levels. And the transcription level of CDKN2A, in turn, correlates with the transcription level of ERCC2.

**Table 3.**
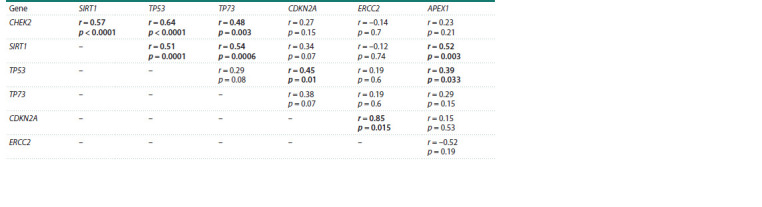
Correlation of genes’ transcription in cervical epithelial cells in the control

At a clinically significant viral load in HPV infection, the dependence of the TP53 and TP73 genes’ transcription and also that of SIRT1 and ERCC2 genes has been shown. The transcription of APEX1 correlated with other genes, except for CDKN2A (Table 4). At the same time, unlike the control, the mRNA level of the CDKN2A gene does not depend on the TP53 gene transcription level (Table 4).

**Table 4.**
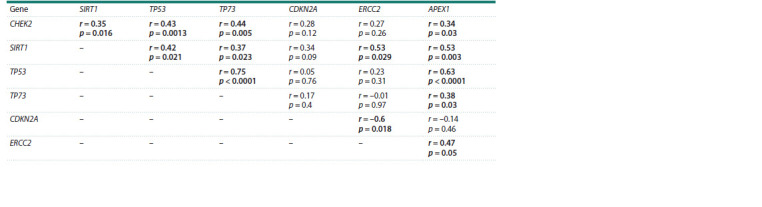
Correlation of genes’ transcription in cervical epithelial cells in HPV-infection

## Discussion

In the work, the level of gene transcription in the cervical canal cells of women over 30 years old in the control group and in the group with an HPV viral load of more than 103 DNA copies of HPV per 105 human cells was investigated. This level of viral load is considered clinically significant and increases the likelihood of dysplastic changes in epithelial cells (AmpliSens… Manual, 2018). The change in the epithelial cell structure is the result of metabolic processes changes in infected cells. The cellular events underlying the transition from normal cervical tissue to cervical dysplasia or cancer at HPV infection remain unexplored. Specifically, it is unknown why some HPV infections persist and progress to cancer, whereas other HPV infections are cleared or precancer tissues return to normal states. In addition, each stage has its own specific HPV-infected epithelial transcription properties.

Cells of the normal cervix highly express multiple tumor suppressors (for example SLC5A8, DERL3), thereby suppressing cell proliferative, migratory and invasive capacities (Guo et al., 2023).

HPV-positive cells with histological changes consistent with CIN I have approximately 20 % of differentially expressed genes compared to normal keratinocytes. Genes of DNA repair and cell cycle (ATM, ATRX) are upregulated. Genes of epithelial differentiation and epidermal development are downregulated. In addition, genes involved in the immune response are downregulated (for example IL-6, STAT1, IFNβ) (Templeton, Laimins, 2024).

At HSIL, high cellular motor capacity is manifested, specifically expressing genes related to cell adhesion (CDH16, CDH17 and VSIG1) and extracellular matrix degradation. It can promote the expansion of atypical cells in intraepithelial neoplasia progression. BRCA1, which plays a crucial role in DNA replication, DNA repair and genomic stability maintenance, is also active in the cells with HSIL. Cancer cells manifest high expression levels of genes related to carcinogenic pathways such as epithelial-to-mesenchymal transition, tumor cell proliferation, migration, invasion and angiogenesis (Guo et al., 2023).

**Fig. 3.**
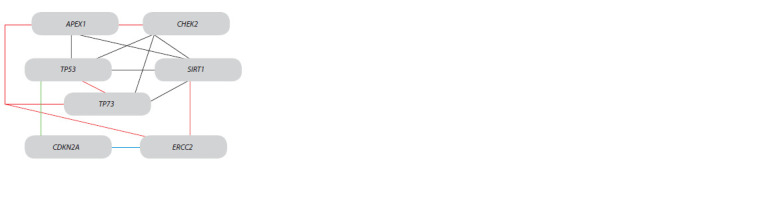
Coexpression of DNA repair and cell cycle control systems genes in cervical epithelial cells. The red lines show genes’ transcription correlation, which was absent in the control; the blue line shows changing directly to negative correlation; the green line – correlation was present only in the control; the black line – correlation was present in both control and HPV infection.

We have evaluated the effect of viral load in HPV infection on the transcription level of the DNA repair and cell cycle control systems genes. A relationship has been revealed between the transcription of the studied genes in uninfected cells. The activity of CHEK2 transcription is associated with the mRNA level of the TP53, TP73, and SIRT1 genes (Table 3, Fig. 3). At the same time, the TP53 gene transcription is associated with the activity of the APEX1 and CDKN2A genes. The last is co-expressed with the ERCC2 gene.

The CHEK2 gene is an oncosuppressor and encodes serine threonine kinase, which reacts to DNA damage and plays a key role in maintaining genome integrity (Bartek et al., 2001). In response to DNA damage, Chk2 kinase is activated, which triggers a protein phosphorylation cascade. The spectrum of phosphorylation substrates includes proteins involved in the cell cycle control, apoptosis and DNA repair, including tumor suppressor p53, cyclin-dependent kinase CDC25C, transcription factors E2F1 and FOXM1, proteins BRCA1 and BRCA2 (Magni et al., 2014; Zannini et al., 2014). In addition, Chk2 participates in the processes of DNA structure modification and cell progression through the cell cycle.

Activation of p53 is also possible under the influence of APEX1. Another protein that participates in the regulation of p53 activity is sirtuin 1 encoded by the SIRT1 gene (Yang et al., 2015; Chen et al., 2021). Sirtuin 1 (SIRT1) is a deacetylase, the activity of which affects gene expression, cell division, and DNA repair

The p14ARF protein takes part in the regulation of p53 degradation processes. p14ARF is one of the transcription products of the CDKN2A gene. This gene encodes two proteins: p16INK4a and p14ARF, which are involved in the regulation of the cell cycle, apoptosis, and cell proliferation (Chan et al., 2021). Protein p16 is a cyclin-dependent kinase inhibitor that is targeted on CDK4 and CDK6 and limits their interaction with cyclin D1 (Giacinti, Giordano, 2006). Protein p14 prevents p53 ubiquitination mediated by ubiquitin-protein ligase E3 MDM2. So, it prevents p53 degradation and ensures p21 expression. The p21 protein inhibits the cyclin/CDK complexes formation (Bieging et al., 2014).

Thus, the coexpression of genes involved in the regulation of the p53 protein was revealed in the cervical epithelial cells of women uninfected by human papillomavirus

In HPV infection, E6 and E7 virus oncoproteins can interact with human proteins of DNA repair and cell cycle control systems. It has been shown that the E6 and E7 proteins interact with CHEK2, which alters its binding efficiency to human DNA and promotes the localization of CHEK2 in HPV DNA replication zones (Gillespie et al., 2012; Bruyere et al., 2023). Also, the E6 protein can bind p53 (Thomas et al., 1999). The decrease in p53 concentration can be partially compensated by p73 activity. p73 activates promoters of several p53-sensitive genes involved in cell cycle control, DNA repair, and apoptosis, and inhibits cell growth in a p53-like manner, inducing apoptosis or cell cycle arrest at the G1 stage (Chellappan et al., 1992; Flores et al., 2002). Our study showed that epithelial cells infected with HPV were characterized by coexpression of the TP53 and TP73 genes, which were absent in control (Fig. 3).

However, the E6 protein is also able to interact with p73. At the same time, the more viral proteins there are, the more pronounced the decrease in p73 activity (Park et al., 2001). We revealed a negative correlation between viral load and the TP53 or TP73 genes transcription level (Table 2). This may serve as a molecular basis for increasing risk of epithelial cell dysplasia and cancer development.

We did not detect changes in gene transcription levels during HPV infection, including those of SIRT1. However, a few studies have shown that the SIRT1 protein level in human cells increases under the influence of the E7 oncoprotein (Allison et al., 2009; Langsfeld et al., 2015). In infected cells, SIRT1 initiates the assembly of a multi-protein viral DNA replication complex (Langsfeld et al., 2015; Das et al., 2019). In addition, SIRT1 can influence the course of the infectious process by regulating the p53 protein level and activity. SIRT1 can bind, and deacetylate activated p53 (Vaziri et al., 2001). We have previously shown that the intergenic interactions of the TP53, TP73, CDKN2A, and SIRT1 genes polymorphic loci affect the risk of clinically significant viral load formation in HPV infection, which may be due to the influence on the cell cycle control and apoptosis processes (AlBosale, Mashkina, 2022; Mashkina et al., 2023).

The literature data on the CDKN2A gene transcription level in carcinogenesis are contradictory. Overexpression of p16INK4a was established in all cervical intraepithelial neoplasm and invasive cervical cancers (Klaes et al., 2001). In several bioinformatic studies analyzing RNA sequencing data of cervical cancer samples, it was found that CDKN2A is a kind of “nodal gene” of the tumor process, since it interacts with various transcription factors, signaling molecules and microRNAs (for example, miR-424-5p and miR-9-5p), and moreover, its overexpression in cervical carcinoma was noted (Zhao et al., 2018). Bioinformatic analysis showed that a change in the CDKN2A gene transcription occurs already at the stage of epithelial cell dysplasia (Kulaeva et al., 2024).

However, at the same time, another study of the CDKN2A expression in cervical cancer cell lines showed that it was reduced; moreover, the authors concluded that CDKN2A inhibits cell proliferation and invasion in cervical cancer through the lactate dehydrogenase-mediated ACT-mTOR pathway (Luan et al., 2021).

We did not detect changes in the CDKN2A transcription level with a significant viral load. However, if in the control, the coexpression of the TP53 and CDKN2A genes transcription levels were revealed, then this dependence disappears in HPV-infected cells. This fact may reflect a change in the p53 protein degradation processes both with the participation of the E6 and E7 proteins, and in a ubiquitin-dependent manner. At the same time, a direct relationship between the mRNA levels of the CDKN2A and ERCC2 genes in the control changes to an inverse relationship at a clinically significant HPV concentration (Table 3). This, on the one hand, may be due to an increase in the number of cells in which the ERCC2 gene is expressed in HPV infection. But, on the other hand, it may be associated with an increase in the amount of DNA damage, which is accompanied by the associated activity of the repair system proteins.

## Conclusion

Thus, an increased frequency of the APEX1 and ERCC2 genes transcription at clinically significant HPV concentrations was established. The reverse dependence of the TP53 and TP73 genes transcription level on the viral load, as well as a change in the coexpression pattern of the studied genes in HPV infection can lead to a change of cell cycle control and apoptosis. These factors can create conditions for the preservation of HPV-infected cells and contribute to an increased risk of epithelial cells’ dysplasia.

## Conflict of interest

The authors declare no conflict of interest.
